# Profiling of human lung and gut microbiomes in different conditions of chronic obstructive pulmonary disease using ontology-based evidence synthesis and reasoning

**DOI:** 10.3389/fcimb.2026.1771765

**Published:** 2026-05-29

**Authors:** Yang Wang, Zeyi Huang, Jie Zheng, Ou Xu, Hong Yu, Xianwei Ye, Yongqun He

**Affiliations:** 1 School of Medicine, Guizhou University, Guiyang, Guizhou, China; 2 Department of Pulmonary and Critical Care Medicine, Guizhou Provincial People’s Hospital, Guiyang, Guizhou, China; 3 College of Literature, Science, and the Arts, University of Michigan, Ann Arbor, MI, United States; 4 Unit for Laboratory Animal Medicine, University of Michigan Medical School, Ann Arbor, MI, United States; 5 Pulmonary Vascular and General Medicine Ward, Fuwai Yunnan Cardiovascular Hospital, Kunming, Yunnan, China; 6 Center for Computational Medicine and Bioinformatics, University of Michigan Medical School, Ann Arbor, MI, United States; 7 Department of Learning Health Sciences, University of Michigan Medical School, Ann Arbor, MI, United States

**Keywords:** COPD, evidence synthesis, gut-lung axis, microbiome & dysbiosis, ontology, ontology-based reasoning, *Prevotella*, Proteobacteria

## Abstract

Chronic Obstructive Pulmonary Disease (COPD) remains one of the leading global causes of morbidity and mortality, with increasing evidence highlighting microbial dysbiosis as a key factor in disease progression and exacerbation. To resolve the inherent heterogeneity in COPD microbiome research, we developed a standardized pipeline termed as Ontology-based Evidence Synthesis and Reasoning (O-ESR), utilizing the Ontology of Host–Microbiome Interactions (OHMI) framework. Our analysis included over 30 studies and identified more than 100 significantly altered bacterial taxa in the human airway and gut microbiomes of human COPD patients across three clinical conditions: COPD versus healthy controls, exacerbation versus stable states, and severe versus moderate diseases. Profiling across taxonomic levels revealed a marked airway expansion of pathogenic genera, including *Haemophilus*, *Moraxella*, *Pseudomonas*, and *Burkholderia*. Species-level analysis confirmed the specific enrichment of *Haemophilus influenzae* and *Pseudomonas aeruginosa*, supporting their roles in airway inflammation and exacerbation susceptibility. In contrast, the gut microbiome of COPD patients exhibited a decrease of beneficial anaerobes involved in short-chain fatty acid (SCFA) production, including *Bifidobacterium bifidum*, *Faecalibacterium prausnitzii*, and members of *Lachnospiraceae* and *Ruminococcaceae*. Notably, ontology-based reasoning identified a shared depletion of commensal genera such as *Prevotella* and *Veillonella* across both anatomical sites and all three clinical conditions, indicating a systemic and progressive loss of microbial diversity. This integrated analysis reveals a COPD-associated microbial landscape characterized by airway Proteobacteria expansion and gut *SCFA-producer* depletion, suggesting coordinated epithelial dysfunction, immune dysregulation, and gut–lung axis involvement. These findings demonstrate the power of ontological reasoning in decoding complex host-microbiome interactions, providing a robust foundation for microbiome-informed stratification and targeted interventions in COPD management.

## 1 Introduction

Chronic Obstructive Pulmonary Disease (COPD) is a major global health burden and ranks as the third leading cause of death worldwide (Wang et al., 2022a). In China alone, COPD accounted for an estimated 50.6 million prevalent cases and 1.29 million deaths in 2021, representing approximately 10.99% of total national mortality (Wang et al., 2022a). Clinically, COPD is characterized by persistent respiratory symptoms and irreversible airflow limitation, frequently accompanied by dyspnea, wheezing, chest tightness, chronic cough with sputum production, recurrent respiratory infections, fatigue, and weight loss (Agustí et al., 2023). COPD is a progressive inflammatory disease primarily caused by long-term exposure to noxious particles or gases, particularly cigarette smoke. The disease involves multifactorial pathophysiological mechanisms, encompassing genetic predispositions, immune dysregulation, and environmental influences. However, the comprehensive picture of the exact mechanisms of COPD is still unclear.

Recent studies have increasingly highlighted the critical role of microbiome in the airways and guts in the pathogenesis and progression of COPD (De Nuccio et al., 2022; Li et al., 2024). The respiratory microbiome, once regarded as passive, is now recognized as a key contributor to COPD onset and progression (Budden et al., 2019; Dicker et al., 2021). In addition to respiratory microbiomes, studies have also shown the close relations between COPD and gut microbiomes (Bowerman et al., 2020; Krumina et al., 2022). Dysbiosis is an imbalance between potentially pathogenic and beneficial microbes in a specific location and has been closely associated with frequent exacerbations and persistent airway inflammation. Dysbiosis of the airway and gut microbial communities has been linked to increased exacerbation frequency, accelerated lung function decline, and systemic inflammation (Hanada et al., 2018; Dicker et al., 2021; Tiew et al., 2021). It is also found that reduced microbial diversity, particularly in the airway microbiome, has been shown to correlate with disease progression, increased exacerbation frequency, and poor clinical outcomes in COPD patients (Molyneaux et al., 2013; Sze et al., 2015). Understanding host–microbiome interactions in the airway and gut of COPD patients therefore offers new opportunities for the rational design of preventive and therapeutic strategies aimed at mitigating disease progression and reducing exacerbations.

With the large number of publications available, evidence-synthesis of the reported results from the literature has become a valuable method for analyzing the profiles of the airway and gut microbiomes under different conditions of COPD patients. To systematically understand the profiles of COPD, we performed a systematical evidence synthesis and analysis by mining, collecting, and categorizing bacterial taxa altered in COPD under three comparative settings: (1) COPD versus healthy controls, (2) exacerbation versus stable disease, and (3) severe versus moderate COPD. This categorization facilitated cross-condition comparisons and highlighted microbial dynamics associated with disease progression and clinical heterogeneity.

To systematically classify and analyze the microbes identified from literature mining, taxonomy and ontology can be used, we leveraged the NCBI taxonomy database (Federhen, 2012; Sakamoto and Ortega, 2021). However, while this database is a standard resource, it is not easy to automatically classify identified microbes at scale. To address this issue, we use the NCBITaxon ontology that maintains the same structure for over 1 million terms in a computer-understandable format. Ontologies support data integration, classification, and semantic reasoning in biomedical informatics (Xiang et al., 2010; Yu et al., 2022). Tools such as OntoFox can extract taxonomic subsets, which can be visualized and edited using platforms like Protégé-OWL. This approach has been widely validated for microbial taxonomic profiling in biomedical research (Moreira and Musen, 2007; Xiang et al., 2010; Musen, 2015).

To further support microbiome knowledge representation and data integration, we have led the development of the community-based Ontology of Host–Microbiome Interactions (OHMI), a logic-driven framework for describing microbial taxa, host entities, interaction types, and experimental contexts (He et al., 2019). OHMI supports consistent annotation and harmonization of host−microbiome data (He et al., 2019). By integrating these ontological tools and frameworks, our study establishes the O-ESR (Ontology-based Evidence Synthesis and Reasoning) methodology. This pipeline systematically transforms unstructured microbial findings from diverse clinical cohorts into a logic-driven, machine-understandable evidence map, enabling more robust knowledge discovery than traditional qualitative reviews.

In this study, we developed and applied the O-ESR framework to decode COPD-associated host–microbe interactions. Our approach identified several novel insights into the COPD microbial landscape, offering a systemic perspective on underlying disease mechanisms across anatomical sites.

## 2 Methods

**Figure 1:**
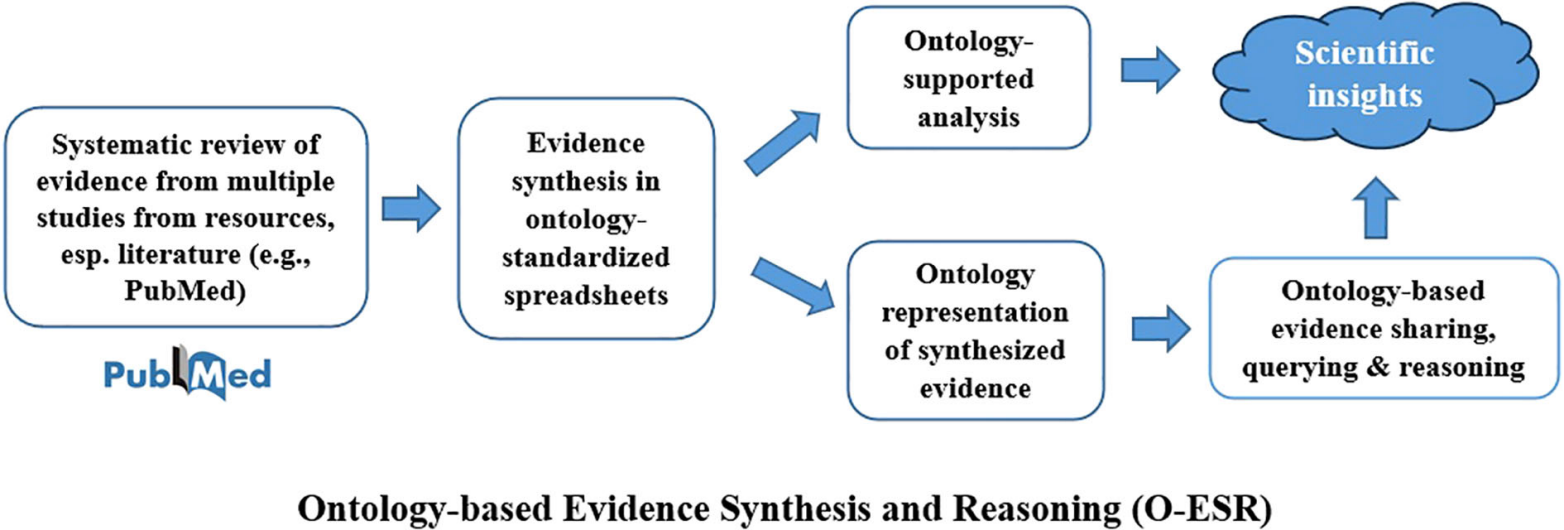
Illustration of the ontology-based evidence synthesis and reasoning (O-ESR) method.

Our ontology-based evidence synthesis and reasoning (O-ESR) method is illustrated in **Figure 1**. Specifically, the method starts with a systematic review and collection of evidence from multiple studies from various resources including the PubMed articles. The evidence is then synthesized in an ontology-standardized spreadsheet(s). The standardized data can then be analyzed using ontology such as the NCBITaxon ontology. Furthermore, the synthesized data can be modeled and represented using an ontology such as the OHMI ontology. With the evidence semantically represented via ontology and stored in an ontological format (such as OWL) and RDF triple store (Ong et al., 2017; He et al., 2019), an ontology-based evidence, sharing, query, and reasoning methods can be used to further uncover specific scientific insights. This approach was used in our COPD-specific study as detailed below.

### 2.1 Systematic literature search and selection criteria

A comprehensive systematic literature search was performed to identify microbial taxa that were significantly increased or decreased in patients with chronic obstructive pulmonary disease (COPD) compared to appropriate controls. As shown in the PRISMA flowchart in **Supplementary Figure 1**, publications were retrieved from the PubMed literature database up to June 2025. For airway COPD related papers, the search keyword terms include: “chronic obstructive pulmonary disease”, “COPD”, “microbiome”, “microbiota”, “airway bacteria”, and “; for gut COPD related papers, keywords including “COPD”, “gut microbiome”, “gut bacteria”, etc.

Inclusion criteria were (1): human studies comparing microbial composition between COPD and non-COPD control groups; (2) studies reporting taxa that were significantly increased or decreased under clearly defined conditions (e.g., stable disease, acute exacerbation, or treatment phase); (3) microbial identification performed using different technologies, including 16S rRNA gene sequencing, quantitative PCR, and 454 pyrosequencing, metagenomics, or culture-based approaches.

Exclusion criteria were: (1) studies without a control group; (2) animal or *in vitro* investigations; (3) studies lacking quantitative microbial abundance data.

Two independent reviewers manually screened the studies and extracted relevant data, and any discrepancies were resolved through consensus. In total, over 30 publications met the inclusion criteria, providing a solid evidence base for subsequent analysis.

### 2.2 Data extraction and taxonomic classification

From the included studies, information on over 100 distinct bacterial taxa was extracted, spanning multiple taxonomic ranks (species, genus, family, order, class, phylum, and kingdom) (Sethi and Murphy, 2008).These taxa were grouped according to three major comparative categories: (1) COPD versus healthy controls, (2) acute exacerbation versus stable COPD, and (3) severe versus moderate COPD.

For each study, patient characteristics (e.g., age, gender, smoking status, pack-years, and lung function indices such as FEV_1_% predicted and FEV_1_/FVC ratio) were recorded to evaluate potential confounders and between-study variability. The FEV_1_/FVC ratio indicates how much air a person can forcefully exhale in one second relative to the total lung capacity. A lower ratio (below 0.7 or 70%) typically signals obstructive lung diseases like COPD.

To ensure taxonomic consistency across studies, each bacterial species was mapped to its NCBI Taxonomy ID using the NIH Taxonomy database (Federhen, 2012). Representative examples include *Bacteroidetes* (Taxon ID: 976), *Prevotella* (ID: 838), *Humicoccus* (ID: 53460), and *Olsenella* (ID: 133925). This mapping enhanced data traceability and ensured uniformity in cross-study comparisons. *“Increased/increase”* and *“decreased/decrease”* were used to describe relative differences in microbial abundance derived from sequencing-based microbiome studies.

All data generated or analyzed during this study are included in this published article and its **Supplementary Information** files. **Supplementary Table 2** contains the master list of respiratory-related studies and their reported taxonomic changes. **Supplementary Table 3** provides gastrointestinal microbiome datasets and their standardized NCBI Taxonomy IDs.

### 2.3 Taxonomical and ontological classification using OntoFox and Protégé

To standardize and hierarchically classify the bacterial taxa, the OntoFox tool (Xiang et al., 2010) was used to extract ontology terms from the NCBI Taxonomy Ontology (NCBITaxon) (Lozano-Fuentes et al., 2013; Wang et al., 2022b). The settings “includeComputedIntermediates” and “includeAllAxioms” were applied to retrieve additional high-level taxonomic terms, ensuring a more comprehensive representation of microbial hierarchy.

The extracted terms were visualized and curated using the Protégé-OWL editor (version 5.6.7) (Musen, 2015). This platform enabled hierarchical organization, refinement of taxonomic relationships, and seamless integration of ontological terms. Through this process, we achieved an accurate and semantically structured classification of the COPD-associated microbiome.

### 2.4 Ontological representation and analysis using OHMI

The relationships between microbes and COPD under different disease conditions were modeled in the Ontology of Host–Microbiome Interactions (OHMI) by following the Open Biomedical Ontology (OBO) foundry principles (Jackson et al., 2021) and eXtensible Ontology Development (XOD) strategy (He et al., 2018) as used in the original OHMI development (He et al., 2019). All annotations were performed using Protégé (v5.6.7) and validated against OHMI’s logical constraints to maintain semantic consistency.

The resulting ontological representation was stored in OWL (Web Ontology Language) format for computational interoperability and further analysis. The code is publicly available at the OHMI GitHub repository (https://github.com/OHMI-ontology/OHMI). The license of access is open access license CC-BY 4.0.

After the OHMI is developed, Description logic (DL) query and SPARQL (i.e., SPARQL Protocol and RDF Query Language) query scripts (Wang et al., 2022; Wang et al., 2024) were both developed to evaluate the usage of OHML for data query and analysis. The OHMI SPARQL scripts are stored in the OHMI GitHub website (https://github.com/OHMI-ontology/OHMI/tree/master/src/sparql). A DL query example is detailed in the Results section.

### 2.5 Ethical considerations

This study was based exclusively on publicly available data and previously published literature. No human participants or animals were directly involved. Therefore, ethical approval and informed consent were not required according to institutional and national research regulations.

## 3 Results

### 3.1 Profiles of COPD microbiomes compared with healthy controls

First of all, we extracted and annotated from the literature bacterial microbiomes in the respiratory system of patients with COPD compared with healthy controls. In total, our literature mining identified many bacterial taxa across multiple taxonomic levels that were either increased or decreased in the respiratory system of patients with COPD as compared with healthy controls (**Table 1**).

**Table 1:** Altered airway bacteria in COPD patients compared to healthy controls.

Bacteria	Taxon level	Taxon ID	PMID
Increased airway bacteria in COPD patients			
*Proteobacteria*	phylum	1224	20052417
*Haemophilus*	genus	724	20052417
*Haemophilus influenzae*	species	727	16474030
*Haemophilus parainfluenzae*	species	729	16474030
*Staphylococcus aureus*	species	1280	16474030
*Streptococcus pneumoniae*	species	1313	16474030
*Pseudomonas aeruginosa*	species	287	16474030
*Neisseriaceae*	family	484	25253795
*Coribacteriales*	order	84999	23071781
*Coriobacteriaceae*	family	84107	23071781
*Atopobium*	genus	1380	23071781
*Cryptobacterium*	genus	84162	23071781
*Olsenella*	genus	133925	23071781
*Nocardioides*	genus	1839	23071781
*Micrococcaceae*	family	1268	23071781
*Rothia*	genus	32207	23071781
*Actinomycetaceae*	family	2049	23071781
*Actinomyces*	genus	1654	23071781
*Bifidobacterium*	genus	1678	23071781
*Lactobacillales*	order	186826	23071781
*Streptococcaceae*	family	1300	23071781
*Streptococcus*	genus	1301	23071781
*Aerococcaceae*	family	186827	23071781
*Abiotrophia*	genus	46123	23071781
*Erysipelotrichales*	order	526525	23071781
*Erysipelotrichaceae*	family	128827	23071781
*Bulleidia*	genus	118747	23071781
*Solobacterium*	genus	123375	23071781
*Veillonellaceae*	family	31977	23071781
*Dialister*	genus	39948	23071781
*Veillonella*	genus	29465	23071781
*Selenomonas*	genus	970	23071781
*Centipeda*	genus	82202	23071781
*Lachnospiraceae*	family	186803	23071781
*Catonella*	genus	43996	23071781
*Oribacterium*	genus	265975	23071781
*Butyrivibrio*	genus	830	23071781
*Eubacteriaceae*	family	186806	23071781
*Eubacterium*	genus	1730	23071781
*Anaerosporobacter*	genus	653683	23071781
*Peptostreptococcus*	genus	1257	23071781
*Parvimonas*	genus	543311	23071781
*Leptotrichiaceae*	family	1129771	23071781
*Leptotrichia*	genus	32067	23071781
*Fusobacteriaceae*	family	203492	23071781
*Fusobacterium*	genus	848	23071781
*Balneimonas*	genus	186650	23071781
*Kingella*	genus	32257	23071781
*Aeromonadales*	order	135624	23071781
*Aeromonadaceae*	family	84642	23071781
*Aeromonas*	genus	642	23071781
*Serratia*	genus	613	23071781
*Desulfobacterales*	order	213118	23071781
*Desulfobulbaceae*	family	213121	23071781
*Desulfobulbus*	genus	893	23071781
*e-proteobacteria*	class	29547	23071781
*Campylobacteriales*	order	213849	23071781
*Campylobacteriaceae*	family	72294	23071781
*Campylobacter*	genus	194	23071781
*Dysgonomonas*	genus	156973	23071781
*Tannerella*	genus	195950	23071781
*Prevotella*	genus	838	23071781
*Hallella*	genus	52228	23071781
*Capnocytophaga*	genus	1016	23071781
*Haemophilus hemolyticus*	species	726	16474030
*Firmicutes*	phylum	1239	28408748
*Lactobacillus*	genus	1578	28408748
*Burkholderia*	genus	32008	22427533
*Pseudomonas*	genus	286	27146202
Decreased airway bacteria in COPD patients			
*Bacteroidetes*	phylum	976	20052417
*Prevotella*	genus	838	20052417
*Arthrobacter*	genus	1663	23071781
*Geodermatophilaceae*	family	85030	23071781
*Nakamurellaceae*	family	85031	23071781
*Modestobacter*	genus	88138	23071781
*Humicoccus*	genus	53460	23071781
*Thermoactinomycetaceae*	family	186824	23071781
*Thermoactinomyces*	genus	2023	23071781
*Clostridium*	genus	1485	23071781
*Citrobacter*	genus	544	23071781
*Prevotellaceae*	family	171552	29992131
*Acidaminococcaceae*	family	909930	29992131

Overall, a total of 69 taxonomic categories, including two phyla (i.e., *Proteobacteria* and *Firmicutes*), one class (i.e., Epsilon *Proteobacteria*), six orders, 15 families, 39 genera, and six species were found to be increased in the airways of COPD patients compared with healthy controls (**Table 1**). For example, members of the phylum *Proteobacteria* (Taxon ID: 1224) were significantly enriched in COPD patients (FEV_1_ ≥95% predicted) compared with healthy controls (Hilty et al., 2010). Within this phylum, the genus *Haemophilus* (Taxon ID: 724) showed increased abundance in COPD subjects (FEV_1_ ≥109% predicted), while other unclassified *Proteobacteria* genera were also elevated (Hilty et al., 2010). At the species level, *Haemophilus influenzae* (Taxon ID: 727), *Pseudomonas aeruginosa* (Taxon ID: 287), *Staphylococcus aureus* (Taxon ID: 1280), *Streptococcus pneumoniae* (Taxon ID: 1313), *Haemophilus haemolyticus* (Taxon ID: 726), and *Haemophilus parainfluenzae* (Taxon ID: 729) were markedly increased in COPD compared with non-smokers (Sethi and Murphy, 2008; Hunt et al., 2020).

Conversely, one phylum (i.e., *Bacteroidetes*), five families, seven genera were found significantly reduced in COPD compared with controls (**Table 1**). The phylum *Bacteroidetes* (Taxon ID: 976) was significantly reduced in COPD compared with controls (FEV_1_ ≥96% predicted). Within this phylum, the genus *Prevotella* (Taxon ID: 838) showed decreased abundance in COPD patients (FEV_1_ ≥103% predicted). The families *Prevotellaceae* (Taxon ID: 171552) and *Acidaminococcaceae* (Taxon ID: 909930) were also diminished, indicating a consistent decline in commensal anaerobes with potential anti-inflammatory roles (Hilty et al., 2010; Pragman et al., 2019).

### 3.2 Microbial composition of COPD patients during exacerbation vs. stable states

A COPD patient’s condition may fluctuate between a stable state and an exacerbation state (Hurst et al., 2010). The COPD exacerbation state represents an acute worsening of respiratory symptoms (e.g., increased breathlessness, cough, and mucus production) beyond stable variations and usually requires a change in medication or hospitalization. We hypothesized that differential microbiome profiles would exist in COPD patients with the two different states. To address this hypothesis, our study examined and identified microbial taxa that were significantly increased- or decreased during exacerbations and could potentially contribute to disease worsening and airway inflammation (**Table 2**).

**Table 2:** Altered COPD airway microbiomes at exacerbation compared with stable situation.

Bacteria	Taxon level	Taxon ID	PMID
Increased bacteria at exacerbation			
*Acinetobacter*	genus	469	24850358
*Perlucidibaca*	genus	661182	24850358
*Actinobacillus*	genus	713	24850358
*Ehrlichia*	genus	943	24850358
*Sphingomonas*	genus	13687	24850358
*Comamonas*	genus	283	24850358
*Enterobacteriaceae*	family	543	24850358
*Pseudomonas*	genus	286	24850358
*Moraxella*	genus	475	26917613
*Haemophilus influenzae*	species	727	15805181
Decreased bacteria at exacerbation			
*Actinobacteria*	phylum	201174	24850358
*Clostridia*	class	186801	24850358
*Bacilli*	class	91061	24850358
*Bacteroidia*	phylum	200643	24850358
*Epsilonproteobacteria*	class	186817	24850358
*Moraxella*	genus	475	24850358, 31234826
*Campylobacter*	genus	194	24850358
*Spirochaetota*	phylum	203691	29386298
*Cardiobacteria*	phylum	95818	31234826
*Dialister*	genus	39948	31234826
*Slackia*	genus	2717	31234826
*Mogibacterium*	genus	86331	31234826
*Treponema*	genus	84108	31234826
*Aggregatibacter*	genus	416916	31234826
*Bulleidia*	genus	118747	31234826
*Megasphaera*	genus	906	31234826
*Prevotella*	genus	838	31234826
*Veillonella*	genus	29465	31234826
*Firmicutes*	phylum	1239	31234826

A total of one family (i.e., *Enterobacteriaceae*), eight genera, and one species (i.e., *Haemophilus influenzae*) were found to have a marked increase in the state of COPD exacerbation compared to its stable state (**Table 2**). Notably, At the class level, multiple exacerbation-enriched taxa mapped to *Gammaproteobacteria*, highlighting this lineage as a major contributor to pathogen-associated dysbiosis during acute episodes (Dima et al., 2019; Dicker et al., 2021). Prominent genera such as *Moraxella*, *Haemophilus*, and *Pseudomonas* exhibited significantly higher relative abundance in exacerbation samples compared with stable conditions (Sethi and Murphy, 2008; López Caro et al., 2019). Specifically, *Moraxella* (Taxon ID: 475) and *Haemophilus* (Taxon ID: 724) demonstrated strong increased, suggesting a potential role in triggering, or worsening respiratory symptoms during acute episodes (Sethi and Murphy, 2008; Huang et al., 2014). Other members of *Gammaproteobacteria*, including *Perlucidibaca* (Taxon ID: 661182) and *Actinobacillus* (Taxon ID: 713), were also more abundant in exacerbation samples.

Conversely, five phyla, three classes, and 11 genera were found to be reduced in the state of COPD exacerbation compared to its stable state (**Table 2**). For example, several bacterial taxa from the *Actinobacteria* phylum, including the families *Microbacteriaceae* and *Nocardiaceae*, were significantly reduced during exacerbation episodes. Similarly, other commensal *Actinobacteria* such as *Rothia* (Taxon ID: 32207) were also less prevalent during exacerbations (Wang et al., 2016). Furthermore, a consistent decrease of *Clostridia* was observed, particularly among *Clostridioides* (Taxon ID: 86331) and *Lachnospiraceae* (Taxon ID: 186803), both of which are typically associated with a stable and balanced airway microbiome (Budden et al., 2019). The decrease of these beneficial anaerobes suggests a loss of microbial homeostasis and metabolic buffering capacity, potentially promoting pro-inflammatory cytokine production and tissue injury (Pragman et al., 2018; Dicker et al., 2021).

Collectively, these findings reveal that COPD exacerbations are characterized by a microbial imbalance, typified by the expansion of potentially pathogenic *Proteobacteria* and the reduction of beneficial *Actinobacteria* and *Clostridia* species. This microbial shift likely contributes to enhanced airway inflammation, impaired immune regulation, and greater clinical severity during exacerbations (Einarsson et al., 2016; Budden et al., 2019). These results underscore the importance of targeting the airway microbiome to prevent and manage COPD exacerbation.

### 3.3 COPD microbiomes in severe patients compared with mild-to-moderate disease

Based on lung function tests (FEV1), symptoms, and exacerbation history, COPD severity is categorized into four stages: mild, moderate, severe, and very severe. The Global Initiative for Chronic Obstructive Lung Disease (GOLD) staging system defined four GOLD levels, which correlate with disease severity including the mild-to-moderate levels (GOLD 1–3) and the severe level (GOLD 4) (Rosemblat et al., 2013). For deep exploration of differential airway microbiome profiles in COPD patients, we examined the altered microbiomes in COPD patients with different severity levels.

As summarized in **Table 3**, one phylum (i.e., *Proteobacteria*), one family (i.e., *Desulfobulbaceae*), and eight genera were found to be increased in the airway microbiomes of severe (GOLD 4) COPD patients compared with mild-to-moderate (GOLD 1–3). Among *Proteobacteria*, the genera *Burkholderia* (Taxon ID 32008) and *Novosphingobium* (Taxon ID 165696) were notably increased in GOLD 4 patients. *Burkholderia* has been frequently isolated from advanced COPD and cystic fibrosis lungs and is associated with antimicrobial resistance and persistent infection (Goldberg et al., 2011). *Novosphingobium* spp. were enriched in GOLD 3–4 COPD groups, possibly reflecting their adaptive capacity in oxidative and low-oxygen niches typical of severe disease. The *Proteobacteria* dominance in severe COPD aligns with findings from Wang et al (Wang et al., 2019). and Dicker et al (Dicker et al., 2021), suggesting that increased *Proteobacteria* abundance may reflect heightened airway inflammation and bacterial colonization associated with advanced airway remodeling. In addition, the genus *Haemophilus* (Taxon ID 724) remained elevated across disease severity levels, indicating its persistent pathogenic role in chronic infection and exacerbation.

**Table 3:** Altered airway microbiomes at severe vs mild-to-moderate COPD patients.

Bacteria	Taxon Level	Taxon ID	PMID
Increased airway bacteria at severe COPD patients			
*Prevotella*	genus	838	27428540
*Acinetobacter*	genus	469	27428540
*Haemophilus*	genus	724	29386298
*Proteobacteria*	phylum	1224	29386298
*Nocardioides*	genus	1839	23071781
*Pontibacillus*	genus	289201	23071781
*Clostridium*	genus	1485	23071781
*Balneimonas*	genus	186650	23071781
*Aeromonas*	genus	642	23071781
*Desulfobulbaceae*	family	213121	23071781
*Desulfobacterales*	order	213118	23071781
*Aeromonadales*	order	135624	23071781
*Aeromonadaceae*	family	84642	23071781
Decreased airway bacteria at severe COPD patients			
*Prevotella*	genus	838	29386298
*Veillonella*	genus	29465	29386298
*Bacteroidetes*	phylum	976	29386298
*Moraxellaceae*	family	468	25253795
*Oribacterium*	genus	265975	25253795
*Eikenella*	genus	538	25253795
*Moraxella*	genus	475	25253795
*Actinobacillus*	genus	713	25253795
*Firmicutes*	phylum	1239	25253795
*Proteobacteria*	phylum	1224	25253795
*Haemophilus parahaemolyticus*	species	735	25253795

Disease severity is classified into three levels: GOLD 4, very severe, moderate.

Meanwhile, three phyla, one family (i.e., *Moraxellaceae*), six genera, and one species (i.e., *Haemophilus parahaemolyticus*), many of which are commensal and anaerobic taxa, were found decreased in the airways of severe COPD patients (**Table 3**). The phylum *Bacteroidetes* (Taxon ID 976) and its representative genus *Prevotella* (Taxon ID 838) showed substantial reduction (Wang et al., 2019). *Prevotella* species are known to contribute to airway immune homeostasis through production of short-chain fatty acids and modulation of host inflammatory pathways; their loss may exacerbate neutrophilic inflammation (Molyneaux et al., 2013). In addition, the genera *Veillonella* (Taxon ID 29465) and *Moraxella* (Taxon ID 475) were consistently reduced in the severe COPD group (Budden et al., 2019; Wang et al., 2019). Although *Moraxella* is typically regarded as a pathogen during acute exacerbations, its lower relative abundance in advanced disease may reflect antibiotic exposure and chronic immune suppression in end-stage COPD.

Overall, severe COPD was characterized by a loss of beneficial anaerobes and enrichment of facultative pathogens, implying that disease progression involves a shift from a balanced commensal microbiota toward a pathogen-dominated ecosystem. This microbial dysbiosis likely perpetuates airway inflammation, disrupts epithelial repair, and promotes a self-sustaining cycle of chronic infection and tissue damage (Budden et al., 2019; Dicker et al., 2021). Both taxonomic diversity and relative abundance patterns suggested that disease severity was associated with selective enrichment of pathogenic taxa and decrease of commensal communities.

### 3.4 Taxonomical and ontological classification of airway microbial composition differences among COPD and other comparative groups

**Figure 2:**
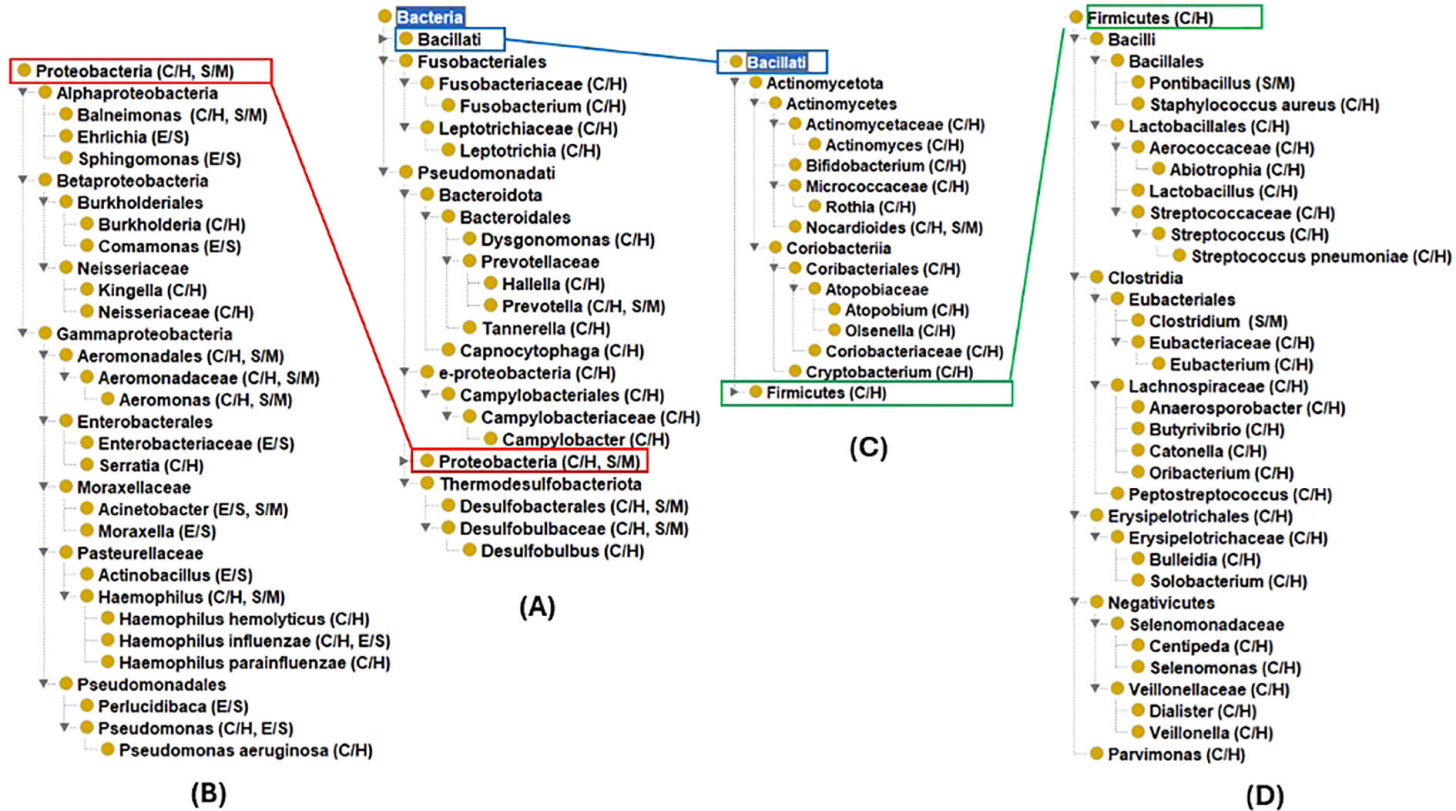
Hierarchical display of increased bacteria of airway microbiota in COPD patients. **(A)** Main hierarchical structure of Bacteria with all layers (except the *Proteobacteria* and *Bacillati* branches) expanded. **(B)** Full expansion of the *Proteobacteria* branch’s hierarchy. **(C)** Full expansion (except *Fimicutes* branch) of the *Bacillati* branch’s hierarchy. **(D)** Full expansion of *Fimicutes* branch’s hierarchy. C/H, COPD vs. healthy controls; E/S, exacerbation vs. stable states; and S/M, severe vs. moderate COPD.

**Figure 3:**
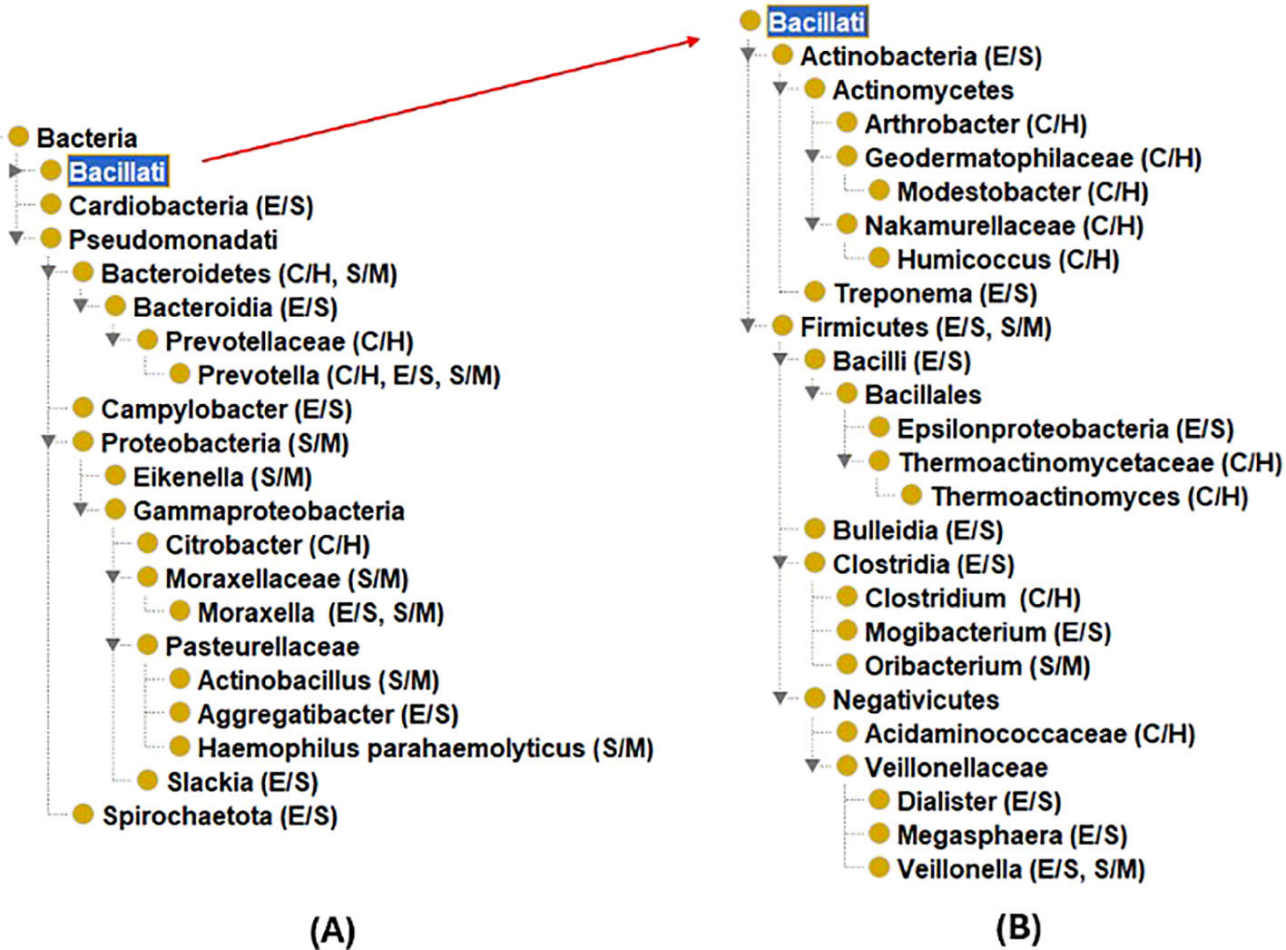
Hierarchical display of decreased bacteria of airway microbiota in COPD patients. **(A)** Main hierarchical structure with all layers except the *Bacillati* branch expanded. **(B)** The full expansion of the *Bacillati* branch’s hierarchy. C/H, COPD vs. healthy controls; E/S, exacerbation vs. stable states; and S/M, severe vs. moderate COPD.

While the above tables summarize differentially abundant taxa across COPD comparisons, they do not capture the hierarchical taxonomic relationships among these microbes. To address this limitation, we converted the lists of differentially regulated taxa into an ontological representation based on the NCBI Taxonomy and visualized the hierarchical structures of increased and decreased airway bacteria in **Figures 2**, **3**, respectively.

**Figure 2** illustrates the hierarchical distribution of increased bacterial taxa that were consistently enriched in COPD across three clinical comparisons: COPD versus healthy controls (C/H), exacerbation versus stable states (E/S), and severe versus moderate disease (S/M). These increased taxa predominantly belonged to three bacterial kingdoms: *Bacillati*, *Fusobacteriati*, and *Pseudomonadati*. Most enriched bacteria under *Bacillati* belong to C/H group. Exceptions to these trends include the genus *Pontibacillus* and *Clostridium*, which were only present in the severe vs. moderate COPD (S/M) comparison, and *Nocardioides*, which appeared in both the COPD vs. healthy controls (C/H) and S/M comparisons. Additionally, *Pseudomonadota* (a subphylum of *Proteobacteria*) includes several bacterial species that were present across all three groups. Overall, the bacterial phyla *Proteobacteria, Pseudomonadota, Actinomycetota*, and *Bacillota* are commonly associated with chronic airway inflammation and disease progression in COPD.

In particular, well-recognized airway pathogens such as *Haemophilus*, *Moraxella*, *Pseudomonas*, and *Aggregatibacter* were enriched during exacerbations (E/S group) and in severe (S/M group) COPD, consistent with previous airway microbiome studies (Hilty et al., 2010; Huang et al., 2014; Wang et al., 2016; Dicker et al., 2021).

**Figure 3** summarizes the hierarchical distribution of bacterial taxa that were consistently decreased in COPD airways. At the *Bacteria* domain level, decreased taxa were mainly distributed within *Bacillati, Cardiobacteria*, and *Pseudomanadati*, indicating that commensal loss in COPD spans multiple phylogenetic lineages rather than a single bacterial group (Erb-Downward et al., 2011; Pragman et al., 2012). Taxa belonging to *Bacillati* and *Pseudomanadati* were observed across all three clinical comparisons, whereas decrease of *Cardiobacteria-associated* taxa was mainly evident in exacerbation-related conditions (Huang et al., 2014; Wang et al., 2016).

At the genus level, *Prevotella* showed consistent decrease across all comparisons (C/H, E/S, and S/M) (Hilty et al., 2010). *Prevotella* was also reported among increased taxa in some studies, reflecting variability in abundance across clinical states (Erb-Downward et al., 2011; Contoli et al., 2017). After deep investigation, we found that these discrepancies were due to multiple reasons including various sampling sites (e.g., sputum, bronchoalveolar lavage, and tissue), sequencing methods, disease stages, smoking status, and antibiotic exposure. For example, *Prevotella* may be more prevalent in early stages of COPD and decline during exacerbations or in severe disease (Hilty et al., 2010; Pragman et al., 2019). *Prevotella* is known to be sensitive to environmental and therapeutic factors as illustrated by the fact that the smoking status and antibiotic use may selectively affect *Prevotella* populations (Erb-Downward et al., 2011; Segal et al., 2016a). In addition, taxa traditionally regarded as airway pathogens, such as *Haemophilus influenzae*, exhibited reduced abundance in severe disease in certain datasets, reflecting non-linear patterns of microbial change with disease progression (Pragman et al., 2012).

Overall, the hierarchical structure reveals substantial overlap between increased and decreased bacterial groups, indicating that COPD-associated airway dysbiosis involves redistribution of microbial taxa rather than uniform loss or gain (Erb-Downward et al., 2011; Segal et al., 2016a).

### 3.5 COPD airway microbiome profiles associated with disease comorbidities

Our research also found that disease comorbidity is a major factor impacting the composition of COPD microbiomes (**Table 4**). Specifically, five bacterial species were found to be increased in COPD patients with specific comorbid conditions. These include *Pseudomonas aeruginosa* and *Haemophilus influenzae*, which were significantly more abundant in COPD patients with bronchiectasis compared to those without. The presence of these pathogens in the COPD microbiome suggests that coexisting bronchiectasis exacerbates the microbial imbalance, leading to an increased burden of respiratory pathogens.

**Table 4:** Altered airway microbiomes in COPD patient with disease comorbidities.

Bacteria	Taxon level	Taxon ID	Change	Control/treatment group	PMID
*Pseudomonas aeruginosa*	Species	287	up	COPD with Bronchiectasis vs Without	23392438
*Haemophilus influenzae*	Species	727	up	COPD with Bronchiectasis vs Without	23392438
*Haemophilus influenzae*	Species	727	up	COPD vs Healthy after rhinovirus infection	23992479
*Proteobacteria*	Phylum	1224	up	COPD vs Healthy after rhinovirus infection	23992479
*Neisseria flavescens*	Species	484	up	COPD on Day 15 after rhinovirus infection vs Baseline	23992479
*Bifidobacterium bifidum*	Species	85004	down	RA patient gut vs Fibromyalgia (FM)	18528968
*Citrobacter*	Genus	544	down	Change in abundance in COPD vs Controls	23071781
*Arthrobacter*	Genus	1663	down	Change in abundance in COPD vs Controls	23071781
*Geodermatophilaceae*	Family	85030	down	Change in abundance in COPD vs Controls	23071781
*Nakamurellaceae*	Family	85031	down	Change in abundance in COPD vs Controls	23071781
*Modestobacter*	Genus	88138	down	Change in abundance in COPD vs Controls	23071781
*Humicoccus*	Genus	53460	down	Change in abundance in COPD vs Controls	23071781
*Thermoactinomycetaceae*	Family	186824	down	Change in abundance in COPD vs Controls	23071781
*Thermoactinomyces*	Genus	2023	down	Change in abundance in COPD vs Controls	23071781
*Anaerobacter (Clostridium)*	Genus	1485	down	Change in abundance in COPD vs Controls	23071781

Additionally, *Proteobacteria* at the phylum level was significantly increased in COPD patients following rhinovirus infection compared to healthy controls. This indicates that viral infections may promote the overgrowth of pathogen-associated bacteria, further contributing to microbial dysbiosis in the airway. *Neisseria flavescens*, also increased in COPD on day 15 post-exacerbation, suggests a temporal shift in bacterial composition following acute flare-ups, which could contribute to the persistence of microbial dysbiosis during recovery phases.

On the other hand, certain genera were decreased in the COPD microbiome, particularly in comparison with healthy controls. For instance, *Bifidobacterium bifidum*, a beneficial gut bacterium, was found to be reduced in COPD patients with comorbidities, particularly in those with rheumatoid arthritis (RA) compared to fibromyalgia (FM) patients. *Furthermore, Citrobacter, Arthrobacter, and Geodermatophilaceae* were decreased in the COPD cohort compared to healthy controls, reinforcing the concept that COPD patients have a distinct microbial profile with a reduced diversity of beneficial bacteria.

These findings underscore the significant role of disease comorbidities in shaping the COPD microbiome, with specific taxa showing either increased or decreased abundance depending on the presence of additional health conditions. The increased abundance of pathogenic bacteria like *Pseudomonas aeruginosa* and *Haemophilus influenzae*, alongside a reduction in beneficial taxa like Bifidobacterium bifidum, highlights the complex interplay between the COPD microbiome and comorbid conditions. This interplay may exacerbate COPD progression and influence disease outcomes.

### 3.6 Increased or decreased gut microbiome profiles of COPD patients

Further, increasing evidence from current research is in favor of the gut–lung axis in COPD, in which intestinal dysbiosis is causally implicated in pulmonary inflammation and disease pathogenesis. In numerous studies, COPD patients demonstrate gut microbiota compositional changes, defined by low abundances of beneficial short-chain-fatty-acid–producing bacteria like *Bifidobacterium* spp.*, Faecalibacterium prausnitzii*, and *Lachnospiraceae* members, concomitantly with high relative abundances of putatively pro-inflammatory taxa like *Streptococcus* and *Prevotella* (Karakasidis et al., 2023; Wang et al., 2023; Budden et al., 2024). These changes in microbes diminish intestinal barrier function, diminish SCFA synthesis, and increase systemic inflammation that could worsen airway damage. Experimental research also shows that intestinal gut microbial balance restoration—by fecal microbiota transfer or dietary complex-carbohydrate treatment—can attenuate lung inflammation in models exposed to cigarettes (Budden et al., 2024). Together, these observations support the idea that gut dysbiosis is pathogenic in COPD through gut–lung immune crosstalk and combating the abnormal balance of microbiota (using methods such as dietary interventions, use of probiotics, and fecal microbial transfer) appears promising in reducing inflammation caused by COPD.

**Figure 4:**
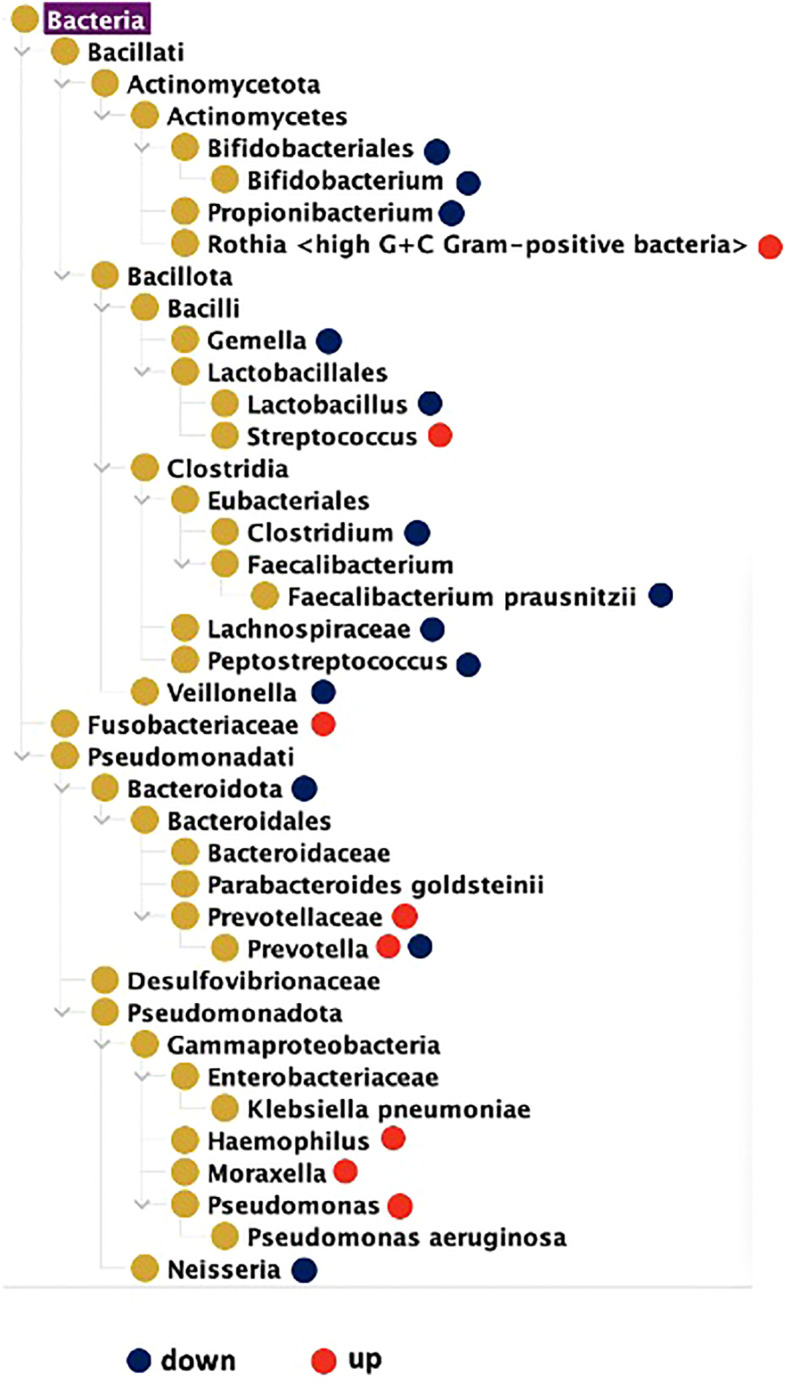
Hierarchical display of phylogenetic distribution of gut microbiota of human COPD patients. Based on **Table 5** collection, both increased and decreased gut bacteria profiles in COPD patients compared with health control are labeled by red dots and blue dots, respectively.

To further analyze the gut profile results, we generated a hierarchical figure using the same method as used for **Figures 2**, **3** (**Figure 4**). Clearly, we can find more information not seen in **Table 5**. For example, the hierarchical classification provides additional insight into the relative abundance of specific bacterial families and genera that may not be captured in the tabular format of **Table 5**. Notably, while **Table 5** highlights the increase of *Proteobacteria* and *Actinobacteria*, the hierarchical figure emphasizes the distinct clustering of these taxa, particularly *Haemophilus, Moraxella*, and *Pseudomonas*, which show strong associations with inflammatory states and disease exacerbation in COPD patients. In contrast, beneficial genera such as *Bifidobacterium* and *Faecalibacterium* are significantly reduced, which may be contributing to the impaired gut barrier function and systemic inflammation observed in these patients.

**Table 5:** Altered bacteria in the gut of human COPD patients.

Bacteria name	NCBI taxon ID	Taxon category	PMID
Increased gut bacteria in COPD patients			
*Proteobacteria*	1224	phylum	37240974
*Actinobacteria*	201174	phylum	37240974
*Haemophilus*	724	genus	37240974; 38778224; 35983167; 37029178
*Moraxella*	475	genus	37240974; 36883945; 38778224; 35983167; 37029178
*Pseudomonas*	286	genus	37240974; 35983167; 37029178
*Enterobacteriaceae*	543	family	37240974
*Streptococcus*	1301	genus	37240974; 36883945; 33208745; 38778224; 35983167
*Desulfovibrionaceae*	194924	family	38331563
*Fusobacteriaceae*	203492	family	36883945
*Prevotellaceae*	171552	family	36883945
*Bacteroidaceae*	815	family	36883945
*Rothia*	32207	genus	33208745
*Klebsiella*	573	genus	37029178
Decreased gut bacteria in COPD patients			
*Bacteroidetes*	976	phylum	37240974; 33687943; 36883945
*Firmicutes*	1239	phylum	37240974; 33687943; 36883945
*Prevotella*	838	genus	37240974; 36883945; 38778224; 37029178
*Veillonella*	29465	genus	37240974; 37029178
*Bifidobacterium*	1678	genus	37240974; 38778224; 35983167
*Faecalibacterium*	216851	genus	37240974
*Parabacteroides goldsteinii*	328812	species	33687943
*Lachnospiraceae*	186803	family	38331563; 36883945; 33208745;​​ 35983167
*Clostridium*	1485	genus	38778224
*Propionibacterium*	1743	genus	38778224
*Lactobacillus*	1578	genus	37029178
*Gemella*	1378	genus	37029178
*Neisseria*	482	genus	37029178

Moreover, the figure visually clarifies the degree of dysbiosis across different disease stages—whether in stable COPD or during acute exacerbations. This enables a clearer understanding of how microbial community shifts align with COPD progression and supports the hypothesis that these microbial shifts could serve as potential biomarkers for disease severity and exacerbation risk. The integration of this hierarchical classification into our analysis also supports the broader concept of the gut-lung axis, suggesting that intestinal microbiome imbalances may exacerbate respiratory inflammation and disease outcomes in COPD.

### 3.7 Leveraging OHMI for the ontological representation and functional analysis of COPD-microbiome interactions

The Ontology of Host-Microbiome Interactions (OHMI) provided a unified semantic framework that allowed us to determine the taxonomic relationships of bacteria at the levels of species, genus, family, and order. The ontological framework also allows for the identification of specific microbial interactions and functional pathways that are potentially involved in the pathogenesis of COPD.

**Figure 5:**
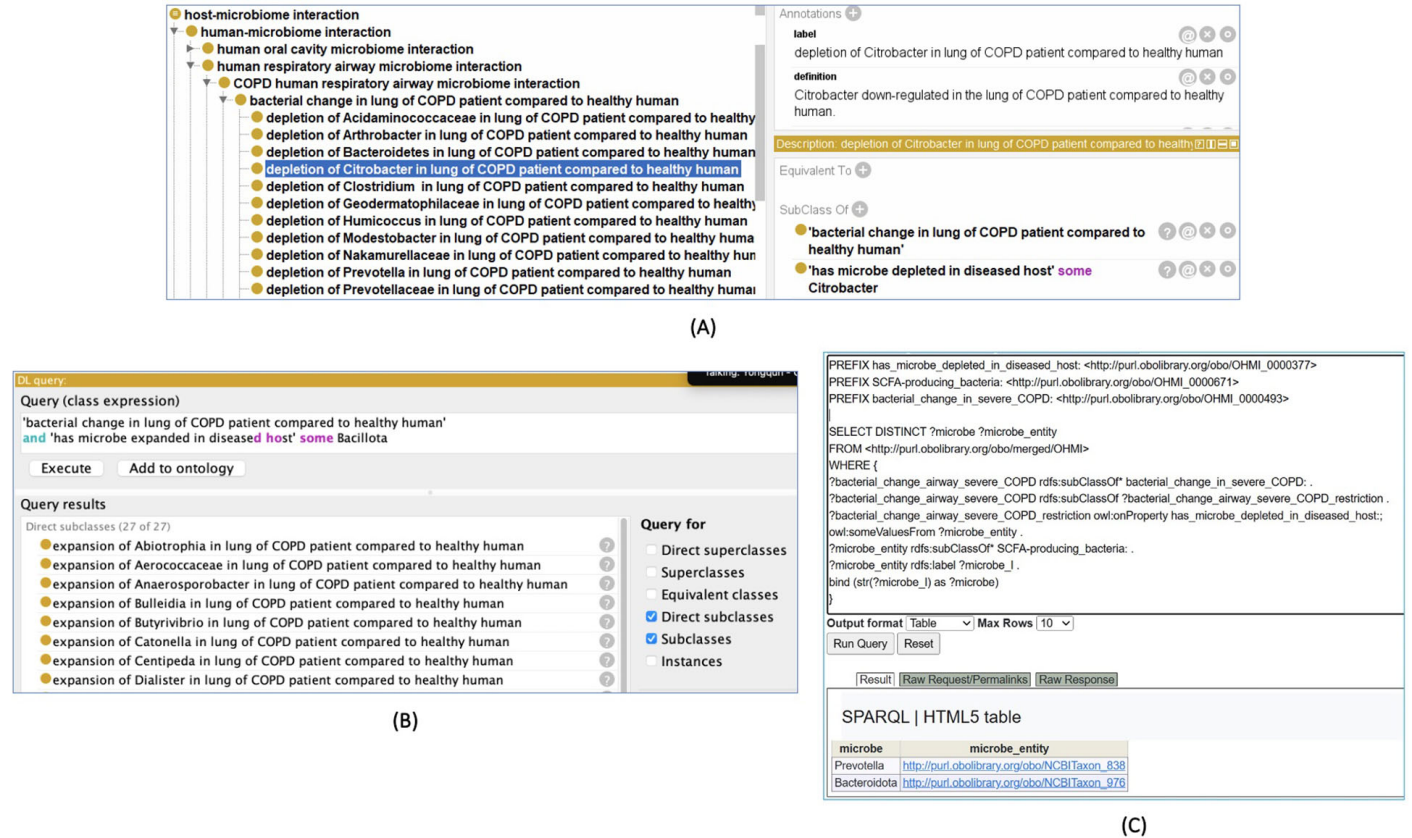
Ontological representation and query example of host-microbiota interactions of human COPD patients. **(A)** An OHMI representation example of ‘decrease of Citrobacter in lung of COPD patient compared to healthy human’. **(B)** A DL-query resulting in the finding of 27 bacteria under the Bacillota that are increased in the lung of COPD patients compared to health control. **(C)** SPARQL query for identifying all SCFA-producing genera depleted in severe COPD.

All the identified COPD-microbiome interactions, including the annotated results shown in the above five tables, were also represented in the OHMI ontology. **Figure 5A** provides an example. Specifically, OHMI represents ‘decrease of *Citrobacter* in lung of COPD patients compared to healthy human’ as a term under the parent class ‘bacterial change in lung of COPD patients compared to healthy human’, which is then further classified as a child term of ‘COPD human respiratory airway microbiome interaction’. In addition to the parent-child relations, OHMI also defines various attributes using ontological axioms For example, the following axiom defines the following the specific relation between ‘decrease of *Citrobacter* in lung of COPD patient compared to healthy human’ and the taxonomical term ‘*Citrobacter*’ (**Figure 5A**):

‘has microbe decreased in disease host’ some Citrobacter.

Furthermore, the knowledge represented in the OHMI ontology can be queried and analyzed using different ways (Wang et al., 2022; Wang et al., 2024). **Figure 5B** provides an example of querying OHMI using DL-query. The specific DL-query script is:

‘bacterial change in lung of COPD patient compared to healthy human’ and ‘has microbe expanded in diseased host’ some Bacillota’.

The above DL-query resulted in 27 hits. The *Bacillota* (synonym *Firmicutes*) are a phylum of bacteria with most having Gram-positive cell wall structure. The above query was able to find many specific bacteria under this phylum, such as genus *Abiotrophia*, *Catonella*, and *Dialister*, which are increased (expanded) in the lungs of COPD patients compared to healthy human controls. The identification of such information relies on the OHMI classification of the taxonomical relations among these taxa based on the NCBI Taxonomy system.

Complementing SPARQL methods (Ong et al., 2017), DL queries leverage the inherent reasoning capabilities of OHMI to uncover functional shifts associated with disease severity. In this study, we utilized an equivalent axiom to define “SCFA-producing bacteria” as any bacterial taxon that participates in the production of short-chain fatty acids (SCFAs). This ontological definition allows the reasoner to automatically infer functional roles across heterogeneous datasets without manual annotation. The DL-query script used was:

‘bacterial change in lung of severe COPD patient compared to mild-moderate COPD’.

‘has microbe depleted in diseased host’ some subClassOf: SCFA-producing bacteria’.

To investigate the functional implications associated with COPD severity, we applied a DL query based on OHMI’s reasoning capabilities. By utilizing an equivalent axiom defining ‘SCFA-producing bacteria’, the reasoner automatically identified 7 taxa significantly depleted in severe COPD compared to mild-moderate stages (**Figure 5C**). These identified taxa included the genera *Prevotella*, *Veillonella*, and *Rothia*, as well as members of the *Lachnospiraceae* family. This ontological approach allowed for the identification of potential functional shifts in the microbial landscape without requiring direct metagenomic data.

## 4 Discussion

Chronic Obstructive Pulmonary Disease (COPD) is a multifactorial disorder with complex microbial dysbiosis playing a central role in disease onset, progression, and exacerbation. In this study, we systematically integrated microbiome data from multiple published studies using the Ontology of Host–Microbiome Interactions (OHMI) framework. This ontology-driven approach enabled standardized representation and comparative evaluation of bacterial taxa associated with COPD under various clinical states.

Our approach is formulated as an “Ontology-based Evidence Synthesis and Reasoning” (O-ESR) framework. Unlike traditional meta-analysis, which focuses on quantitative aggregation and statistical comparison across studies, O-ESR emphasizes structured evidence collection and ontology-informed synthesis and analysis. Rather than performing pooled statistical analysis, our method applies qualitative synthesis supported by semantic ontologies, followed by formal ontology-based representation and computational analysis of the integrated evidence. In our study, conclusions are derived using systematic approaches such as vote-counting (e.g., the number of studies reporting enrichment versus depletion), instead of estimating effect sizes or statistical significance. The O-ESR framework is summarized from many similar studies by us and others in different biomedical domains (Dissanayake et al., 2020; He et al., 2020; Wang et al., 2021; Wang et al., 2022; Wang et al., 2024). While the absence of quantitative meta-analysis may limit certain forms of statistical inference, O-ESR offers several complementary advantages. First, ontology guidance enables consistent data standardization and classification across heterogeneous studies. Second, the framework includes modular, machine-interpretable ontological representation of synthesized evidence, facilitating data sharing and reuse. Finally, ontology-based knowledge representation enables advanced semantic querying and reasoning over the integrated evidence, as demonstrated in this work. The application of the O-ESR framework has proven successful in our COPD-specific study.

Our findings identified more than 100 significantly altered bacterial taxa in the airway and gut microbiomes of human COPD patients across three clinical comparisons: COPD patients versus healthy controls, COPD exacerbation versus stable states, and severe versus moderate COPD conditions. COPD patients exhibited many increased profiles. For example, compared to health controls, we found the enrichment of *Proteobacteria*, particularly pathogenic genera such as *Haemophilus*, *Pseudomonas*, and *Moraxella*, which is a hallmark of COPD-associated dysbiosis (Sethi and Murphy, 2008; Budden et al., 2019). As disease severity increases (e.g., GOLD 4), we observed further significant enrichment of *Firmicutes* and *Proteobacteria*, where chronic inflammation and antibiotic exposure favor the expansion of pro-inflammatory and opportunistic taxa (Wang et al., 2019; Dicker et al., 2021). Notably, acute exacerbations (AECOPD) are characterized by the acute surge of *Gammaproteobacteria*, particularly *Moraxella catarrhalis*, *Haemophilus influenzae*, and *Pseudomonas aeruginosa* (Murphy et al., 2004; Wang et al., 2016). These organisms, frequently detected in sputum during exacerbations, are known to trigger pro-inflammatory cytokine release (e.g., IL-8 and TNF-α) and align with a surge in bacterial load in neutrophilic endotypes (Mayhew et al., 2018; Dicker et al., 2021). The regulatory direction of certain taxa, such as *Moraxella*, varied across studies, likely reflecting differences in sampling site, disease stage, antibiotic exposure, and exacerbation definition. Collectively, the overrepresentation of these pathogenic *Proteobacteria* across different conditions supports the hypothesis that dysbiosis drives chronic airway inflammation and susceptibility to exacerbation (Molyneaux et al., 2013; Huang et al., 2014; Budden et al., 2019; Dicker et al., 2021).

Many decreased profiles in COPD patients were also identified. Compared to healthy controls, we found a consistent reduction in commensal taxa such as *Prevotella*, *Veillonella*, and *Lachnospiraceae* in COPD patients, particularly in advanced disease stages where microbial diversity tends to decrease. In severe COPD conditions, *Bacteroidetes* and *Actinobacteria* were significantly diminished (Rosemblat et al., 2013; Dicker et al., 2021). Furthermore, compared to stable states, exacerbations are associated with a profound ecological disruption marked by the further decrease of beneficial commensals, such as *Clostridia*, *Rothia*, and *Actinobacteria*. These beneficial taxa, especially members of *Lachnospiraceae*, play critical roles in maintaining immune homeostasis by producing short-chain fatty acids (SCFAs) and suppressing pro-inflammatory signaling (Budden et al., 2019). The systemic loss of these SCFA-producers may amplify inflammatory responses and lead to tissue injury during exacerbations, indicating that such episodes represent more than transient infections but a fundamental shift in the respiratory and potentially gut microbiome (Einarsson et al., 2016).

**Figure 6:**
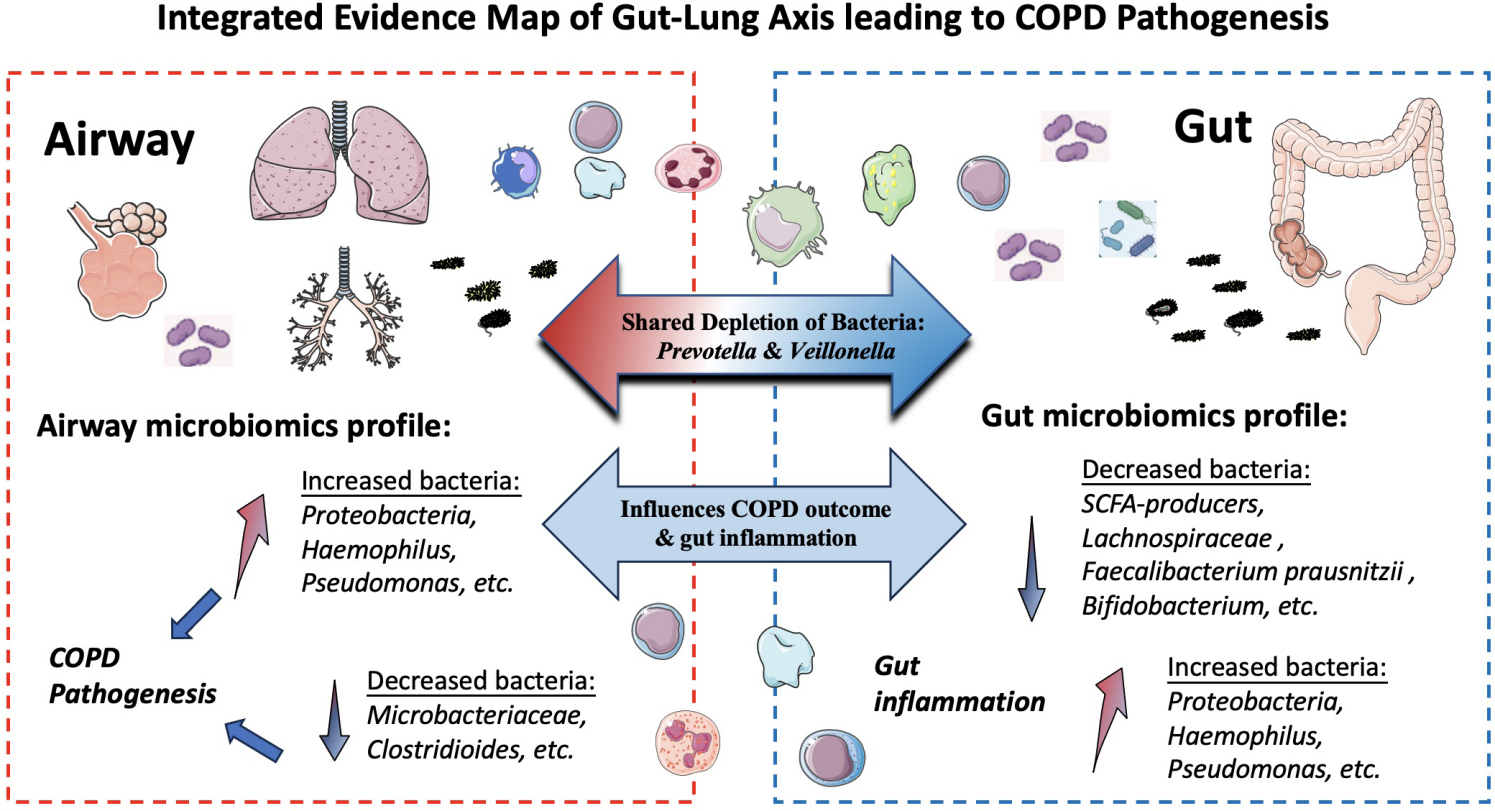
Diagram of the integrated evidence map of the gut-lung axis in COPD patients.

Our O-ESR analysis of the lung and gut microbiome profiles supports the broader paradigm of the gut-lung axis, a distributed immune-metabolic network that significantly influences the COPD pathogenesis and outcomes (**Figure 6****).** In addition to the different microbial enrichment or depletion listed in the figure and described above, the intestinal microbiome imbalances out of the gut dysbiosis may result in the imbalance of metabolites such as short-chain fatty acids (SCFA) (Jiang et al., 2023; Tanabe et al., 2025), which will further exacerbate respiratory inflammation and COPD disease outcomes. SCFAs (e.g., acetate, propionate, and butyrate) promote regulatory T cells (Tregs), reduce Th17-mediated inflammation, and enhance antiviral responses (Segal et al., 2016b; Jiang et al., 2024). SCFAs circulate systemically and can influence lung macrophages, dendritic cells, and epithelial responses. Respiratory infections such as influenza and COVID-19 can alter gut microbiome composition and increase gut inflammation, which may further affect airway condition and COPD pathogenesis. The bi-directionary feedback loop can amplify COPD disease severity. Further studies are needed to investigate the immune-mediated mechanisms and microbial metabolites that link gut dysbiosis to pulmonary inflammation, which would lead to advanced rational preventative and therapeutic product design.

Using the NCBITaxon and OHMI ontologies, the ontology-based evidence synthesis and reasoning (O-ESR) framework was proven valuable in identifying COPD-related microbiomics profiles and uncovering scientific insights. The NCBITaxon ontology-based classification allows us to systematically classify the identified bacteria effectively and robustly. Meanwhile, the modeling and representation of the synthesized evidence to the OHMI ontology make the results standardized and accessible for future machine processing and easy reuse. New methods such as the description logic (DL) query and SPARQL query can be applied for advanced analysis. While inter-study heterogeneity exists due to varying sampling methods, our ontology-based integration identified a consistent core signature that transcends individual study biases. Furthermore, other ontology-driven artificial intelligence (AI) approaches, such as ontology-based knowledge graph development and graph neural network (Zheng and He, 2025) can be used to support more advanced analysis, predictions, and clinical decision-making processes.

Despite the advancements made in this study, there are limitations to consider. First, most included studies relied on 16S rRNA sequencing, which has limitations in taxonomic resolution and functional inference. Second, variability in sampling sites (e.g., sputum, bronchoalveolar lavage, or tissue) and patient characteristics (e.g., smoking history, comorbidities, and antibiotic use) may introduce bias. Third, our analysis focused primarily on bacterial communities, while fungal and viral microbiota (Hanada et al., 2018; Gaibani et al., 2021), which also influence COPD pathogenesis, remain underexplored. Furthermore, the keyword searching strategy used in this study might not be inclusive and might have missed some important published articles in specific areas such as gut-lung axis in COPD. To address this issue, we may need to test more variable keywords and possibly use the-state-of-the-art large language model (LLM) methods (Li et al., 2024; Wang et al., 2025).

Many directions exist for future studies. In science, it is critical to integrate multi-omics data, including metagenomics, transcriptomics, metabolomics, and host immune profiling, to establish causal relationships between microbial dysbiosis and disease mechanisms. Longitudinal studies with standardized metadata will be crucial to distinguish transient from persistent microbial changes. We can also hypothesize that the enriched microbes in COPD patients contain genes and proteins that act as virulence factors. It is possible to use the “reverse microbiomics” strategy to compare the genomes of enriched and declined microbes to identify possible virulence factors (Wang et al., 2021; Wang et al., 2022b). Furthermore, it is possible to rebalance the loss of beneficial microbes and maintain microbial diversity to mitigate disease progression. In informatics, the OHMI ontology and the O-ESR framework can be further developed and applied. We will continuously accumulate and represent synthesized evidence related to COPD and other diseases. We also plan to systematically define, standardize, and enhance the framework for more advanced usage. The O-ERS framework can also incorporate more ontology-based common data models such as the Study-Experiment-Assay Common Data Model (SEA CDM) (Huffman et al., 2026). Furthermore, we think this framework is applicable for other biomedical studies. 

## Data Availability

The original contributions presented in the study are included in the article/**Supplementary Material**. Further inquiries can be directed to the corresponding authors.

## References

[B1] Agustí A. Celli B. R. Criner G. J. Halpin D. Anzueto A. Barnes P. . (2023). Global initiative for chronic obstructive lung disease 2023 report: GOLD executive summary. Eur. Respir. J. 61 (4), 2300239. doi: 10.1183/13993003.00239-2023 36858443 PMC10066569

[B2] Bowerman K. L. Rehman S. F. Vaughan A. Lachner N. Budden K. F. Kim R. Y. . (2020). Disease-associated gut microbiome and metabolome changes in patients with chronic obstructive pulmonary disease. Nat. Commun. 11, 5886. doi: 10.1038/s41467-020-19701-0. PMID: 33208745 PMC7676259

[B3] Budden K. F. Shukla S. D. Bowerman K. L. Vaughan A. Gellatly S. L. Wood D. L. A. . (2024). Faecal microbial transfer and complex carbohydrates mediate protection against COPD. Gut 73, 751–769. doi: 10.1136/gutjnl-2023-330521. PMID: 38331563

[B4] Budden K. F. Shukla S. D. Rehman S. F. Bowerman K. L. Keely S. Hugenholtz P. . (2019). Functional effects of the microbiota in chronic respiratory disease. Lancet Respir. Med. 7, 907–920. doi: 10.1016/s2213-2600(18)30510-1. PMID: 30975495

[B5] Contoli M. Pauletti A. Rossi M. R. Spanevello A. Casolari P. Marcellini A. . (2017). Long-term effects of inhaled corticosteroids on sputum bacterial and viral loads in COPD. Eur. Respir. J. 50 (4), 1700451. doi: 10.1183/13993003.00451-2017. PMID: 28982774

[B6] De Nuccio F. Piscitelli P. Toraldo D. M. (2022). Gut–lung microbiota interactions in chronic obstructive pulmonary disease (COPD): potential mechanisms driving progression to COPD and epidemiological data. Lung 200, 773–781. doi: 10.1007/s00408-022-00581-8. PMID: 36241745

[B7] Dicker A. J. Huang J. T. J. Lonergan M. Keir H. R. Fong C. J. Tan B. . (2021). The sputum microbiome, airway inflammation, and mortality in chronic obstructive pulmonary disease. J. Allergy Clin. Immunol. 147, 158–167. doi: 10.1016/j.jaci.2020.02.040. PMID: 32353489

[B8] Dima E. Kyriakoudi A. Kaponi M. Vasileiadis I. Stamou P. Koutsoukou A. . (2019). The lung microbiome dynamics between stability and exacerbation in chronic obstructive pulmonary disease (COPD): current perspectives. Respir. Med. 157, 1–6. doi: 10.1016/j.rmed.2019.08.012. PMID: 31450162

[B9] Dissanayake P. I. Colicchio T. K. Cimino J. J. (2020). Using clinical reasoning ontologies to make smarter clinical decision support systems: a systematic review and data synthesis. J. Am. Med. Inform Assoc. 27, 159–174. doi: 10.1093/jamia/ocz169. PMID: 31592534 PMC6913230

[B10] Einarsson G. Comer D. McIlreavey L. Parkhill J. Ennis M. Tunney M. . (2016). Community dynamics and the lower airway microbiota in stable chronic obstructive pulmonary disease, smokers and healthy non-smokers. Thorax 71, 795–803. doi: 10.1136/thoraxjnl-2015-207235. PMID: 27146202

[B11] Erb-Downward J. R. Thompson D. L. Han M. K. Freeman C. M. McCloskey L. Schmidt L. A. . (2011). Analysis of the lung microbiome in the “healthy” smoker and in COPD. PloS One 6, e16384. doi: 10.1164/ajrccm-conference.2010.181.1_meetingabstracts.a5628. PMID: 21364979 PMC3043049

[B12] Federhen S. (2012). The NCBI taxonomy database. Nucleic Acids Res. 40, D136–DD43. doi: 10.1093/nar/gkr1178. PMID: 22139910 PMC3245000

[B13] Gaibani P. Viciani E. Bartoletti M. Lewis R. E. Tonetti T. Lombardo D. . (2021). The lower respiratory tract microbiome of critically ill patients with COVID-19. Sci. Rep. 11, 10103. doi: 10.1038/s41598-021-89516-6. PMID: 33980943 PMC8115177

[B14] Goldberg J. B. Ganesan S. Comstock A. T. Zhao Y. Sajjan U. S. (2011). Cable pili and the associated 22 kDa adhesin contribute to Burkholderia cenocepacia persistence *in vivo*. PloS One 6, e22435. doi: 10.1371/journal.pone.0022435. PMID: 21811611 PMC3141045

[B15] Hanada S. Pirzadeh M. Carver K. Y. Deng J. C. (2018). Respiratory viral infection-induced microbiome alterations and secondary bacterial pneumonia. Front. Immunol. 9. doi: 10.3389/fimmu.2018.02640. PMID: 30505304 PMC6250824

[B16] He Y. Wang H. Zheng J. Beiting D. P. Masci A. M. Yu H. . (2019). OHMI: the ontology of host-microbiome interactions. J. Biomed. Semant 10, 25. doi: 10.1186/s13326-019-0217-1. PMID: 31888755 PMC6937947

[B17] He Y. Xiang Z. Zheng J. Lin Y. Overton J. A. Ong E. (2018). The eXtensible ontology development (XOD) principles and tool implementation to support ontology interoperability. J. Biomed. Semant 9, 3. doi: 10.1186/s13326-017-0169-2. PMID: 29329592 PMC5765662

[B18] He Y. Yu H. Ong E. Wang Y. Liu Y. Huffman A. . (2020). CIDO, a community-based ontology for coronavirus disease knowledge and data integration, sharing, and analysis. Sci. Data 7, 181. doi: 10.2139/ssrn.5344128. PMID: 32533075 PMC7293349

[B19] Hilty M. Burke C. Pedro H. Cardenas P. Bush A. Bossley C. . (2010). Disordered microbial communities in asthmatic airways. PloS One 5, e8578. doi: 10.1371/journal.pone.0008578. PMID: 20052417 PMC2798952

[B20] Huang Y. J. Sethi S. Murphy T. Nariya S. Boushey H. A. Lynch S. V. (2014). Airway microbiome dynamics in exacerbations of chronic obstructive pulmonary disease. J. Clin. Microbiol. 52, 2813–2823. doi: 10.1128/jcm.00035-14. PMID: 24850358 PMC4136157

[B21] Huffman A. Yeh F. Y. Hur J. Zheng J. Masci A. M. Wu G. . (2026). SEA CDM: Study-Experiment-Assay common data model and databases for cross-domain data integration and analysis. Sci. Data 13, 238. doi: 10.1038/s41597-026-06558-z. PMID: 41535301 PMC12905146

[B22] Hunt B. C. Stanford D. Xu X. Li J. Gaggar A. Rowe S. M. . (2020). Haemophilus influenzae persists in biofilm communities in a smoke-exposed ferret model of COPD. ERJ Open Res. 6 (3), 00200–2020. doi: 10.1183/23120541.00200-2020. PMID: 32802827 PMC7418822

[B23] Hurst J. R. Vestbo J. Anzueto A. Locantore N. Müllerova H. Tal-Singer R. . (2010). Susceptibility to exacerbation in chronic obstructive pulmonary disease. N. Engl. J. Med. 363, 1128–1138. doi: 10.1056/nejmoa0909883. PMID: 20843247

[B24] Jackson R. Matentzoglu N. Overton J. A. Vita R. Balhoff J. P. Buttigieg P. L. . (2021). OBO Foundry in 2021: operationalizing open data principles to evaluate ontologies. Database (Oxford) 2021, baab069. doi: 10.1093/database/baab069. PMID: 34697637 PMC8546234

[B25] Jiang M. Li Z. Zhang F. Li Z. Xu D. Jing J. . (2023). Butyrate inhibits iILC2-mediated lung inflammation via lung-gut axis in chronic obstructive pulmonary disease (COPD). BMC Pulm Med. 23, 163. doi: 10.1186/s12890-023-02438-z. PMID: 37173731 PMC10182695

[B26] Jiang M. Wang J. Li Z. Xu D. Jing J. Li F. . (2024). Dietary fiber-derived microbial butyrate suppresses ILC2-dependent airway inflammation in COPD. Mediators Inflamm. 2024, 6263447. doi: 10.1155/2024/6263447. PMID: 39015676 PMC11251798

[B27] Karakasidis E. Kotsiou O. S. Gourgoulianis K. I. (2023). Lung and gut microbiome in COPD. J. Pers Med. 13, 804. doi: 10.3390/jpm13050804. PMID: 37240974 PMC10221978

[B28] Krumina A. Bogdanova M. Gintere S. Viksna L. (2022). Gut–lung microbiota interaction in COPD patients: a literature review. Medicina 58, 1760. doi: 10.3390/medicina58121760. PMID: 36556962 PMC9785780

[B29] Li R. Li J. Zhou X. (2024). Lung microbiome: new insights into the pathogenesis of respiratory diseases. Signal Transduct Target Ther. 9 (1), 19. doi: 10.1038/s41392-023-01722-y. PMID: 38228603 PMC10791971

[B30] Li X. Zheng Y. Hu J. Zheng J. Wang Z. He Y. (2024). VaxLLM: Leveraging fine-tuned large language model for automated annotation of Brucella vaccines. bioRxiv. doi: 10.1101/2024.11.25.625209. PMID: 39651132 PMC11623542

[B31] López Caro J. C. Santibáñez M. García Rivero J. L. Villanueva M. Sainz J. González Astorqui P. . (2019). Sputum microbiome dynamics in chronic obstructive pulmonary disease patients during an exacerbation event and post-stabilization. Respiration 98, 447–454. doi: 10.1159/000501988. PMID: 31437842

[B32] Lozano-Fuentes S. Bandyopadhyay A. Cowell L. G. Goldfain A. Eisen L. (2013). Ontology for vector surveillance and management. J. Med. Entomol. 50, 1–14. doi: 10.1603/me12169. PMID: 23427646 PMC3695545

[B33] Mayhew D. Devos N. Lambert C. Brown JR. Clarke SC. Kim VL. . (2018). Longitudinal profiling of the lung microbiome in the AERIS study demonstrates repeatability of bacterial and eosinophilic COPD exacerbations. Thorax 73 (5), 422–430. doi: 10.1136/thoraxjnl-2017-210408 29386298 PMC5909767

[B34] Molyneaux P. L. Mallia P. Cox M. J. Footitt J. Willis-Owen S. A. Homola D. . (2013). Outgrowth of the bacterial airway microbiome after rhinovirus exacerbation of chronic obstructive pulmonary disease. Am. J. Respir. Crit. Care Med. 188, 1224–1231. doi: 10.1164/rccm.201302-0341oc. PMID: 23992479 PMC3863728

[B35] Moreira D. A. Musen M. A. (2007). OBO to OWL: a protege OWL tab to read/save OBO ontologies. Bioinformatics 23, 1868–1870. doi: 10.1093/bioinformatics/btm258. PMID: 17496317

[B36] Murphy T. F. Brauer A. L. Schiffmacher A. T. Sethi S. (2004). Persistent colonization by Haemophilus influenzae in chronic obstructive pulmonary disease. Am. J. Respir. Crit. Care Med. 170, 266–272. doi: 10.1164/rccm.200403-354oc. PMID: 15117742

[B37] Musen M. A. (2015). The protégé project: a look back and a look forward. AI Mattr 1 (4), 4–12. doi: 10.1145/2757001.2757003 PMC4883684 27239556

[B38] Ong E. Xiang Z. Zhao B. Liu Y. Lin Y. Zheng J. . (2017). Ontobee: a linked ontology data server to support ontology term dereferencing, linkage, query and integration. Nucleic Acids Res. 45, D347–DD52. doi: 10.1093/nar/gkw918. PMID: 27733503 PMC5210626

[B39] Pragman A. A. Kim H. B. Reilly C. S. Wendt C. Isaacson R. E. (2012). The lung microbiome in moderate and severe chronic obstructive pulmonary disease. PLoS One 7 (10), e47305. doi: 10.1371/journal.pone.0047305 23071781 PMC3469539

[B40] Pragman A. A. Knutson K. A. Gould T. J. Isaacson R. E. Reilly C. S. Wendt C. H. (2019). Chronic obstructive pulmonary disease upper airway microbiota alpha diversity is associated with exacerbation phenotype: a case-control observational study. Respir. Res. 20, 114. doi: 10.1186/s12931-019-1080-4. PMID: 31174538 PMC6555967

[B41] Rosemblat G. Resnick M. P. Auston I. Shin D. Sneiderman C. Fizsman M. . (2013). Extending semRep to the public health domain. J. Am. Soc Inf. Sci. Technol. 64, 1963–1974. doi: 10.1002/asi.22899. PMID: 24729747 PMC3981099

[B42] Sakamoto T. Ortega J. M. (2021). Taxallnomy: an extension of NCBI Taxonomy that produces a hierarchically complete taxonomic tree. BMC Bioinf 22, 388. doi: 10.1186/s12859-021-04304-3. PMID: 34325658 PMC8323199

[B43] Segal L. N. Alekseyenko A. V. Clemente J. C. Kulkarni R. Wu B. Gao Z. . (2016a). Enrichment of the lung microbiome with oral taxa is associated with lung inflammation. Nat. Microbiol. 1, 16031. doi: 10.1038/nmicrobiol.2016.31. PMID: 27572644 PMC5010013

[B44] Segal L. N. Clemente J. C. Tsay J. C. Koralov S. B. Keller B. C. Wu B. G. . (2016b). Enrichment of the lung microbiome with oral taxa is associated with lung inflammation of a Th17 phenotype. Nat. Microbiol. 1, 16031. doi: 10.1038/nmicrobiol.2016.31. PMID: 27572644 PMC5010013

[B45] Sethi S. Murphy T. F. (2008). Infection in the pathogenesis and course of chronic obstructive pulmonary disease. N. Engl. J. Med. 359, 2355–2365. doi: 10.1056/nejmra0800353. PMID: 19038881

[B46] Sze M. A. Dimitriu P. A. Suzuki M. McDonough J. E. Campbell J. D. Brothers J. F. . (2015). Host response to the lung microbiome in chronic obstructive pulmonary disease. Am. J. Respir. Crit. Care Med. 192, 438–445. doi: 10.1164/rccm.201502-0223oc. PMID: 25945594 PMC4595667

[B47] Tanabe N. Matsumoto H. Morimoto C. Hirai T. (2025). Sputum short-chain fatty acids, microbiome, inflammation, and mucus plugging in obstructive airway disease. J. Allergy Clin. Immunol. 155, 1675–1680. doi: 10.1016/j.jaci.2025.01.031. PMID: 39914553

[B48] Tiew P. Y. Jaggi T. K. Chan L. L. Chotirmall S. H. (2021). The airway microbiome in COPD, bronchiectasis and bronchiectasis‐COPD overlap. Clin. Respir. J. 15, 123–133. doi: 10.1111/crj.13294. PMID: 33063421

[B49] Wang L. Cai Y. Garssen J. Henricks P. A. J. Folkerts G. Braber S. (2023). The bidirectional gut–lung axis in chronic obstructive pulmonary disease. Am. J. Respir. Crit. Care Med. 207 (9), 1145–1160. doi: 10.1164/rccm.202206-1066TR. PMID: 36883945 PMC10161745

[B50] Wang Z. Bafadhel M. Haldar K. Spivak A. Mayhew D. Miller B. E. . (2016). Lung microbiome dynamics in COPD exacerbations. Eur. Respir. J. 47, 1082–1092. doi: 10.1183/13993003.01406-2015. PMID: 26917613

[B51] Wang Z. Li X. Zheng J. He Y. (2025). Unveiling differential adverse event profiles in vaccines via LLM text embeddings and ontology semantic analysis. J. Biomed. Semant 16, 10. doi: 10.1186/s13326-025-00331-8. PMID: 40410898 PMC12102970

[B52] Wang Z. Maschera B. Lea S. Kolsum U. Michalovich D. Van Horn S. . (2019). Airway host-microbiome interactions in chronic obstructive pulmonary disease. Respir. Res. 20, 113. doi: 10.1186/s12931-019-1085-z. PMID: 31170986 PMC6555748

[B53] Wang H. Ong E. Kao J. Y. Sun D. He Y. (2021). Reverse Microbiomics: A new reverse dysbiosis analysis strategy and its usage in prediction of autoantigens and virulent factors in dysbiotic gut microbiomes from rheumatoid arthritis patients. Front. Microbiol. 12. doi: 10.3389/fmicb.2021.633732. PMID: 33717026 PMC7947680

[B54] Wang Y. Ye M. Zhang F. Freeman Z. T. Yu H. Ye X. . (2024). Ontology-based taxonomical analysis of experimentally verified natural and laboratory human coronavirus hosts and its implication for COVID-19 virus origination and transmission. PloS One 19, e0295541. doi: 10.1371/journal.pone.0295541. PMID: 38252647 PMC10802970

[B55] Wang H. Ye X. Zhang Y. Ling S. (2022a). Global, regional, and national burden of chronic obstructive pulmonary disease from 1990 to 2019. Front. Physiol. 13. doi: 10.3389/fphys.2022.925132. PMID: 36017339 PMC9396373

[B56] Wang Y. Zhang F. Byrd J. B. Yu H. Ye X. He Y. (2022). Differential COVID-19 symptoms given pandemic locations, time, and comorbidities during the early pandemic. Front. Med. (Laus) 9. doi: 10.3389/fmed.2022.770031. PMID: 35155491 PMC8831795

[B57] Wang H. Zhang K. Wu L. Qin Q. He Y. (2022b). Prediction of pathogenic factors in dysbiotic gut microbiomes of colorectal cancer patients using reverse microbiomics. Front. Oncol. 12. doi: 10.3389/fonc.2022.882874. PMID: 35574378 PMC9091335

[B58] Xiang Z. Courtot M. Brinkman R. R. Ruttenberg A. He Y. (2010). OntoFox: web-based support for ontology reuse. BMC Res. Notes 3, 175. doi: 10.1186/1756-0500-3-175. PMID: 20569493 PMC2911465

[B59] Yu H. Li L. Huffman A. Beverley J. Hur J. Merrell E. . (2022). A new framework for host-pathogen interaction research. Front. Immunol. 13. doi: 10.3389/fimmu.2022.1066733. PMID: 36591248 PMC9797517

[B60] Zheng Y. He Y. (2025). VaxjoGNN: A graph neural network for ontologygrounded vaccine adjuvant recommendation. bioRxiv. doi: 10.1101/2025.11.27.690985

