# Supplementary material for: Profiling of human lung and gut microbiomes in different conditions of chronic obstructive pulmonary disease using ontology-based evidence synthesis and reasoning
Source: Front Cell Infect Microbiol. 2026 May 29;16:1771765. doi: 10.3389/fcimb.2026.1771765 (PMC13259719; doi:10.3389/fcimb.2026.1771765)
Supplement: Supplementary file 1 [file Table1.docx]

**Supplemental Figure 1. PRISMA flowchart:**

58,426 records related to airway COPD identified by searching PubMed up to May 2021 using keywords including: “Chronic Obstructive Pulmonary Disease”, ”COPD”, ”microbiome”, ”microbiota”, and ”airway bacteria”, etc.

6,763 records related to gut COPD identified from searching PubMed up to June 2025 using keywords including ”COPD”, “gut microbiome” ,“gut bacteria”, etc.

**Identification**

Records excluded:

(1) Studies without a control group;

(2) Animal or in vitro investigations; (3) Studies lacking quantitative microbial abundance data;

(4) Not COPD-related;

(5) Reviews or editorials.

(n = 61,407)

**Screening**

Records after duplicates removed (n = 3,782)

Full-text articles excluded:

no differential taxa reported; Inadequate control/comparator; Insufficient data.

(n =1,822).

Full-text articles assessed

for eligibility (n = 1,960)

**Eligibility**

Studies included in qualitative synthesis (n = 41, including

33 from airway COPD,

and 8 from gut COPD)

**Included**

**Supplemental Figure 1. PRISMA flowchart for the study selection process of the systematic review and meta-analysis conducted on COPD-related microbiome studies**. A comprehensive search strategy was employed across PubMed, identifying a total of 58,426 records related to COPD, microbiome, airway bacteria, and the gut-lung axis up until June 2025. After the removal of duplicates, 3,782 records remained for screening. The title and abstract screening process resulted in the exclusion of 61,407 records, primarily due to the following reasons: lack of control group, animal or in vitro studies, studies that did not report quantitative microbial data, and articles that were not related to COPD or the microbiome (e.g., reviews, editorials). Following the screening stage, 1,960 full-text articles were assessed for eligibility. Of these, 1,822 articles were excluded based on predefined criteria, including inadequate taxonomic analysis or irrelevant study designs. Ultimately, 41 studies were included in the qualitative synthesis and were subjected to ontology-based analysis, including microbial taxonomy integration via the Ontology of Host–Microbiome Interactions (OHMI) framework. The flowchart visually summarizes the process, ensuring transparency and reproducibility in the review methodology.
